# The Shining Star of the Last Decade in Regional Anesthesia Part-II: Interfascial Plane Blocks for Cardiac, Abdominal, and Spine Surgery

**DOI:** 10.5152/eurasianjmed.2023.23015

**Published:** 2023-12-01

**Authors:** Ahmet Murat Yayık, Erkan Cem Çelik, Muhammed Enes Aydın, Elif Oral Ahıskalıoğlu, Burhan Dost, Başak Altıparmak, Madan Narayanan, Alessandro De Cassai, Serkan Tulgar, Ali Ahıskalıoğlu

**Affiliations:** 1 Department of Anaesthesiology and Reanimation, Atatürk University School of Medicine, Erzurum, Turkey; 2 Clinical Research, Development and Design Application and Research Center, Atatürk University School of Medicine, Erzurum, Turkey; 3 Department of Anaesthesiology and Reanimation, On Dokuz Mayıs University School of Medicine, Istanbul, Turkey; 4 Department of Anaesthesiology and Reanimation, Sıtkı Koçman University School of Medicine, Muğla, Turkey; 5 Department of Anaesthesia, Frimley Health NHS Foundation Trust, Frimley, UK; 6 Department of Anesthesia and Intensive Care, Padua University Hospital, Padua, Italy; 7 Department of Anesthesiology and Reanimation, Samsun University Faculty of Medicine, Samsun, Turkey

**Keywords:** Interfascial plane block, acute pain, multimodal analgesia, postoperative analgesia, cardiac, abdominal, and spine surgeries

## Abstract

The sine qua non of enhanced recovery after surgery protocols designed to improve the perioperative experiences and outcomes of patients is to determine the most appropriate analgesia management. Although many regional techniques have been tried over the years in this purpose, interfacial plane blocks have become more popular with the introduction of ultrasound technology into daily practice and they have great potential to support effective postoperative pain management in many surgeries. The current article focuses on the benefits, techniques, indications, and complications of interfascial plane blocks applied in cardiac, abdominal, and spine surgeries.

**Main Points**

- The sine qua non of enhanced recovery after surgery protocols designed to improve the perioperative experiences and outcomes of patients is to determine the most appropriate analgesia management.
- Interfacial plane blocks have become more popular with the introduction of ultrasound technology into daily practice.
- Plane blocks have great potential to support effective postoperative pain management in many surgeries.

## Introduction

Interfascial plane blocks have been used in many surgeries in recent years, especially with the widespread use of ultrasound in anesthesia practice. These blocks are a regional anesthesia technique that involve the injection of local anesthetics into the space between the 2 fascia layers rather than trying to locate a particular nerve or plexus.^1^ The erector spinae plane (ESP) block, transversus abdominis plane block, and quadratus lumborum block are prominent among these blocks.^2^ A PubMed search revealed over 1127 papers on ESP block as of December 2022. This popularity can be attributed to its ease of application and safe and effective analgesia.

Up to 30%-80% of individuals may experience pain ranging from moderate to severe on the first postoperative day.^3^ Uncontrolled acute postoperative pain can lead to chronic pain, decreased quality of life, and an increased risk of pulmonary complications. Although neuraxial techniques are used for pain relief after surgery, they have some serious drawbacks, including an increased risk of epidural/spinal hematoma, potential hemodynamic instability, technical difficulties, and pneumothorax.^4^ Enhanced Recovery After Surgery (ERAS) protocols are increasingly being used in cardiac, abdominal, and spine procedures. These opioid-sparing techniques reduce opioid requirements and length of stay and are associated with significantly improved perioperative outcomes.^5^

This review aims to provide an overview of interfascial plane blocks used for perioperative analgesia in cardiac, abdominal, and spine surgeries.

## Cardiac Surgery

One of the main causes of death worldwide is cardiovascular disease, which accounts for around one-third of all mortality.^6^ There will probably be a rise in the number of cardiothoracic surgeries due to the aging population in the United States.^7^

For a patient to recuperate fully from surgery, the pain from their median sternotomy must be managed as best as possible. Serious side effects that may develop due to inadequate pain management include respiratory failure,^8^ increased myocardial work and oxygen demand,^9^ delayed mobilization,^10^ and longer hospital stays.^10,11^ Additionally, higher postoperative pain scores are related to a greater prevalence of chronic pain syndromes.^12^

Open heart surgery was made easier with the introduction of high-dose intravenous morphine in the late 1960s.^13^ Early in the 1990s, long-acting high-dose opioid techniques were utilized to reduce sympathetic response to surgical pain and enhance hemodynamic stability.^13^ However, despite the fact that they are related to undesirable side effects such as nausea, vomiting, somnolence, and constipation, opioids have become a common component of perioperative therapy. While they allow for proper surgical exposure with keeping a stable hemodynamic profile, they have forced patients to endure prolonged postoperative mechanical breathing and lengthy recovery times, despite the fact that they offer short-term benefits.

Contrary to colorectal or outpatient surgery, where the negative consequences of opioid usage are more obvious,^14^ cardiac surgery adopted the Enhanced recovery after surgery principles later because the community took longer to catch on to the benefits of its use.^15^ Despite the rapid advantages in analgesia protocols, postoperative pain management for heart surgery patients still routinely involves large opioid dosages (>10-15 mcg/kg per patient).^16^ Recent research suggests that more than 30% of the cardiac surgery patients experience a potentially opioid-related adverse event during hospitalization,^17^ in contrast to much lower rates in the non-cardiac population. Also that shows more than 15% of opioid-naive patients continue to take opioids 90-120 days after a hospital stay.^18-20^ In order to dramatically minimize to dependence on opioid-based protocols during heart surgery, it is imperative to develop new techniques.

Opioid-free and opioid-sparing analgesia procedures have recently become more popular as a result of their benefits in preventing opioid-related side effects, which have been demonstrated.^17,21^ In order to give an alternative to conventional opioid-dominated analgesia regimens utilized during cardiac surgery, regional analgesia approaches are being used more frequently.^4^

In recent years, interfascial plane blocks have become an essential component of multimodal analgesia protocols, especially in cardiovascular surgery, due to their high safety profile. Superficial and deep parasternal intercostal plane (PIP) block,^22,23^ serratus anterior plane (SAP) block,^24^ interpectoral plane (IPP) and pectoserratus plane (PSP) block,^25^ and erector spinae plane (ESP) block^26^ will be discussed in this review.

### Superficial and Deep Parasternal Intercostal Plane Blocks

These blocks performed in the parasternal region are called superficial or deep parasternal intercostal plane blocks, depending on whether the local anesthetic injection is applied to the surface or deep of the intercostal muscle.^2^ Both blocks involve the anterior branches of the intercostal nerves in the T2–T6 dermatomes, which are responsible for the innervation of the sternum.^27,28^

**Figure 1. A-D.**
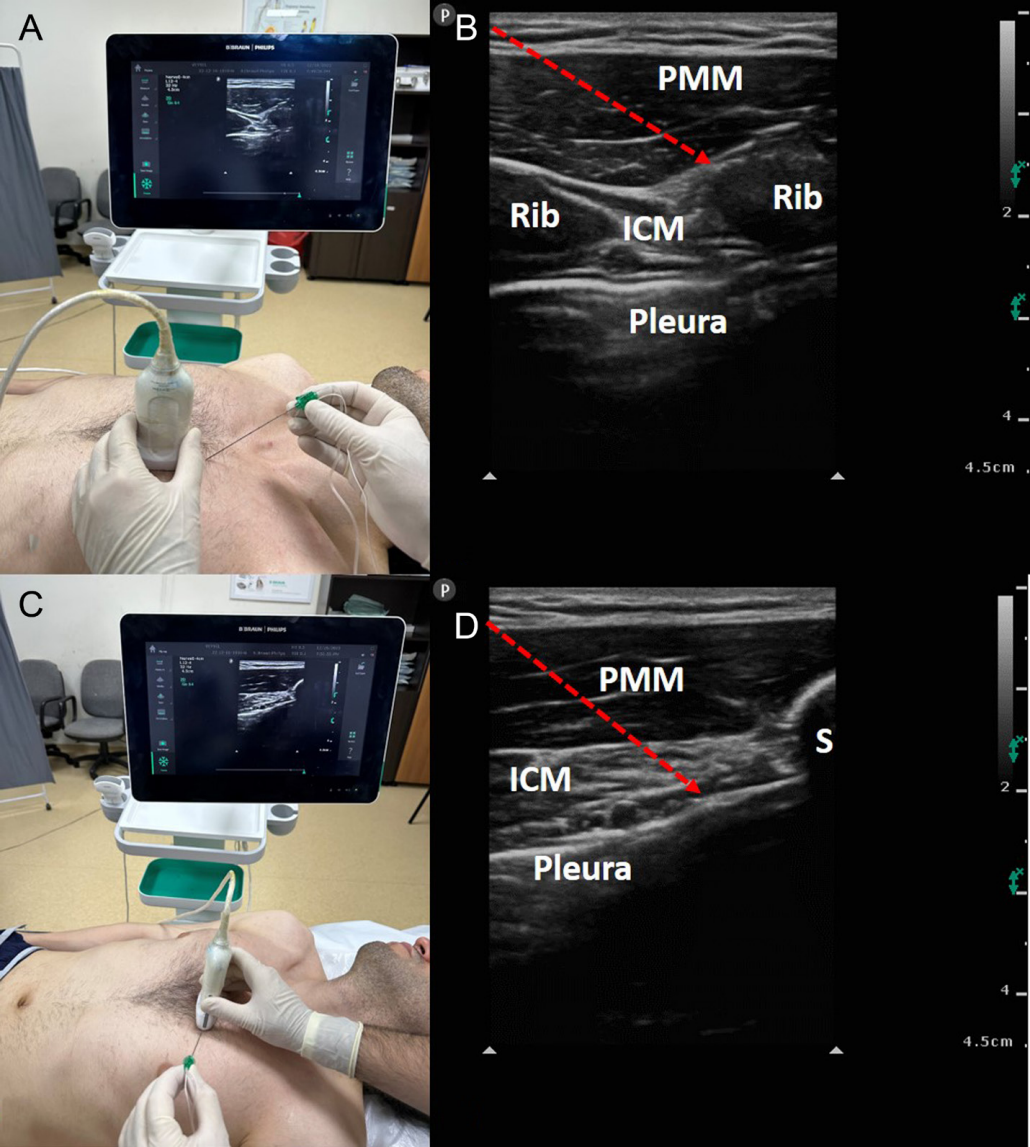
Patient and ultrasound probe position for superficial (A) and deep (C) parasternal intercostal plane blocks procedure. (B, D) Sonographic anatomy of the block. ICM, intercostal muscles; PMM, pectoralis major muscle;red arrow, needle; S, sternum.

Parasternal blocks can be performed in the supine position before or after the incision. In the superficial PIP block procedure, the ultrasound probe is parasagittally placed 1 cm lateral to the sternal border (Figure 1A). Between the fourth and fifth ribs, the parasternal sagittal view of the pectoralis major muscle, internal intercostal muscle, and transversus thoracis muscle is visualized above the pleura. After excluding intravascular interference to the plane between the pectoralis major muscle and the internal intercostal muscles, the injection is administered with intermittent aspirations (Figure 1B). Caudo-cranial spread may be observed simultaneously with the injection.

In the deep PIP block procedure, the linear ultrasound probe is transversely placed 1 cm lateral to the sternal border (Figure 1C). Between the fourth and fifth ribs, the transverse view of the pectoralis major muscle, internal intercostal muscle, and transversus thoracic muscle is visualized above the pleura. Besides, imaging the internal mammary artery and vein and performing the procedure from lateral to medial is critical to minimize the possible risk of vascular puncture. After excluding intravascular interference to the plane between the internal intercostal muscle and the transversus thoracis muscles (Figure 1D), the injection is administered with intermittent aspirations. With the injection, the transversus thoracis muscle is confirmed by the spread of local anesthetic both adjacent to the internal intercostal muscle and adjacent to the endothoracic fascia just below it.

Randomized and observational studies of superficial and deep PIP blocks in median sternotomy have demonstrated a consistent association between both improved pain scores and reduced opioid consumption compared to control groups.^29,30^ In addition to its analgesic effectiveness, deep PIP block application has been reported as an anesthetic method for sternal revision in high-risk patients.^31^ There are new findings in the literature that hydro dissection around the internal mammary artery may have facilitating effects in surgical harvesting in deep PIP blocks.^32^ A randomized clinical study comparing superficial and deep PIP blocks in cardiac surgery revealed that both blocks had similar effects on 24-hour morphine consumption and pain scores.^33^ More studies are required to evaluate the efficacy of deep PIP block, which has been demonstrated to be effective in chronic pain with a case report and compared it to other methods.^34^

### Interpectoral Plane and Pectoserratus Plane Blocks

These blocks performed on the anterior chest wall are called interpectoral or PSP blocks, depending on whether the local anesthetic injection is applied to the surface or deep of the pectoralis minor muscle. These pectoral region blocks include T3-T6 intercostal nerves, medial-lateral pectoral nerves, intercostobrachial, and long thoracic nerves.^35,36^

Parasternal blocks can be performed in the supine position before or after the incision.

**Figure 2. A,B.**
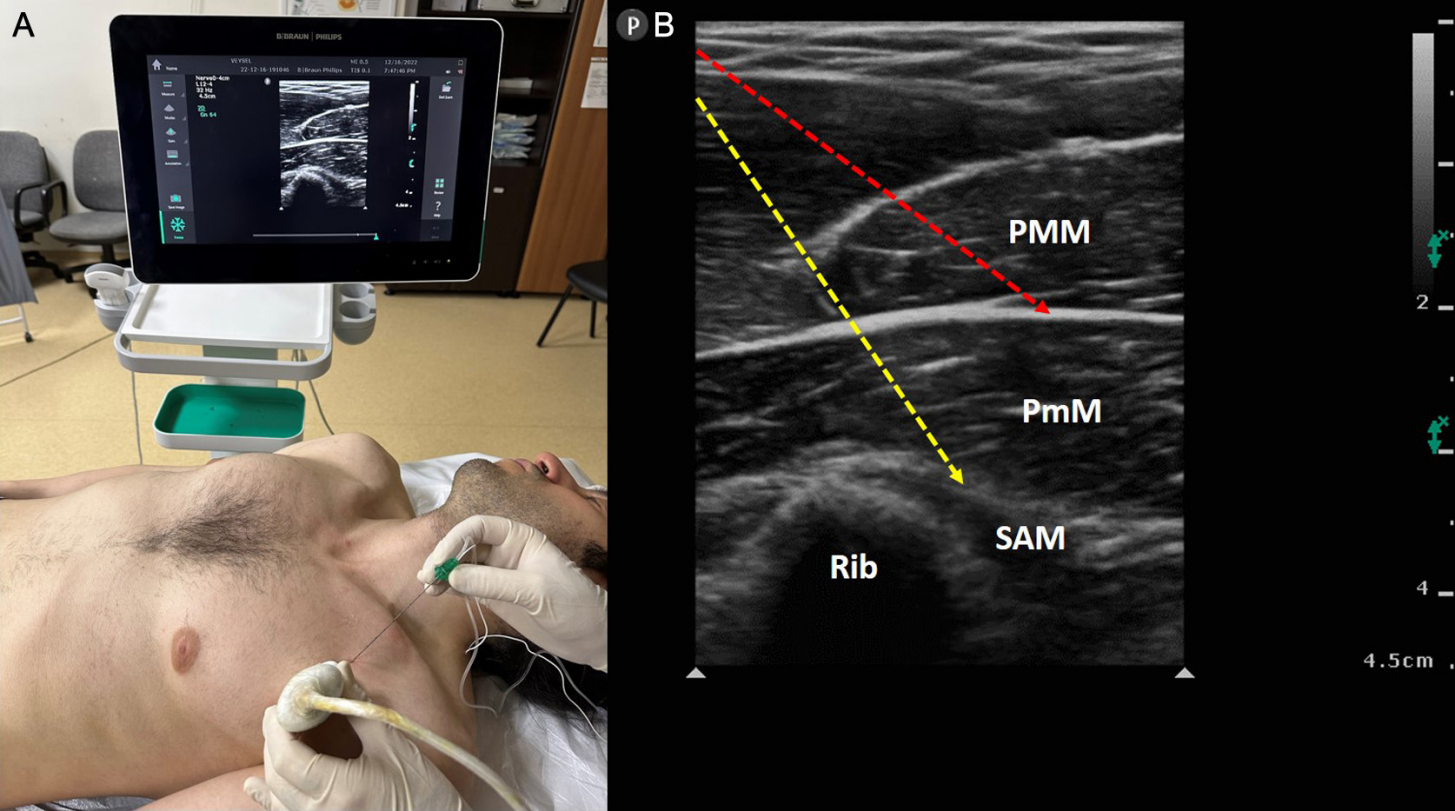
(A) Patient and ultrasound probe position for interpectoral plane block and pectoserratus plane block procedure. (B) Sonographic anatomy of the block. PMM, pectoralis major muscle; PmM,pectoralis minor muscle; red arrow, needle direction for interpectoral plane block; SAM, serratus anterior muscle; yellow arrow, needle direction for pectoserratus plane block procedure.

In the block procedure, the linear ultrasound probe is placed longitudinally in the subclavian region with the axillary artery visible. It is advanced caudally to the axillary artery, then shifted laterally to view the second rib, then caudally to observe the third and fourth ribs (Figure 2A). The pectoralis major, pectoralis minor, and serratus anterior muscles are visualized. The IPP block is performed by the injection of local anesthetic between the pectoralis major and pectoralis minor muscle, and the PSP block is performed with local anesthetic injection between the pectoralis minor muscle and serratus anterior muscle (Figure 2B).

These blocks may be preferred to relieve pain in the anterior chest wall. Forty patients who were having valve operations or coronary artery bypass grafting (CABG) by midline sternotomy were randomly assigned to the postoperative Pectoralis (PECS) (IPP and PSP) block group or the no block group by Kumar et al.^37^ When compared to no block group, the block group was extubated much sooner. At 0, 3, 6, 12, and 18 hours following extubation, the block group had reduced scores for pain while at rest and coughing. The block group also had greater peak inspiratory flow rates measured by incentive spirometry. In a case who underwent mitral valve repair surgery using the right anterior thoracotomy technique, Yalamuri et al^38 ^documented a case in which IPP and PSP blocks were used as rescue analgesia. The block used 30 mL of 0.20% ropivacaine and 1 : 400 000 epinephrine to almost completely anesthetize the chest wall.

### Superficial and Deep Serratus Anterior Plane Blocks

These blocks, which are performed on the side wall of the chest, are called superficial or deep serratus anterior plane blocks, depending on whether the local anesthetic injection is applied to the surface or deep of the serratus anterior muscle.^2^ The serratus anterior plane block covers the lateral cutaneous branches of the intercostal nerves in the T3-T9 range.^39^

Serratus anterior plane blocks can be performed in the supine position before or after the incision.

**Figure 3. A,B.**
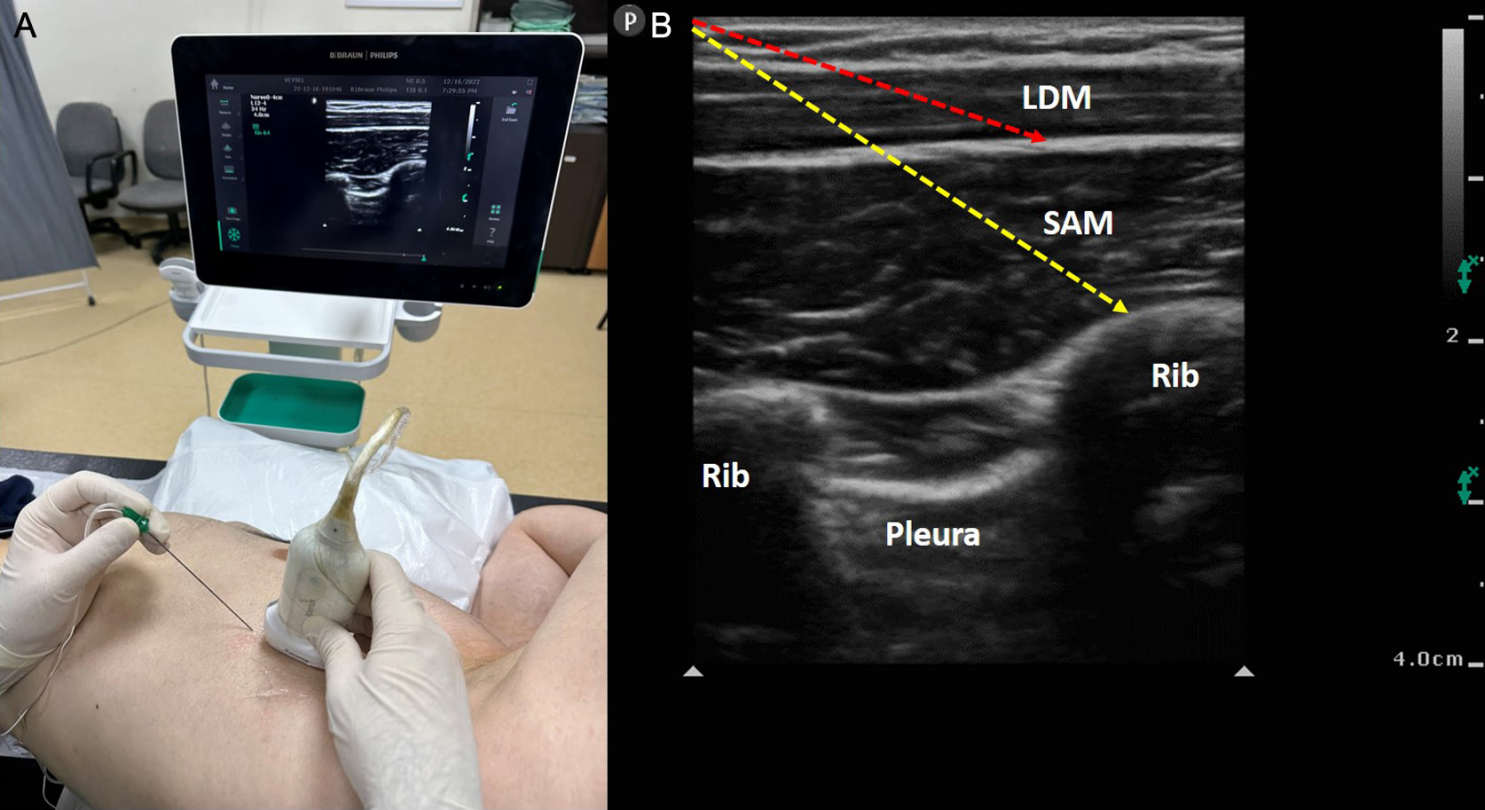
(A) Patient and ultrasound probe position for serratus anterior plane block procedure. (B) Sonographic anatomy of the block. LDM, latissimus dorsi muscle; red arrow, needle direction for superficial serratus anterior plane block; SAM, serratus anterior muscle; yellow arrow, needle direction for deep serratus anterior plane block.

In the serratus anterior plane block procedure, the linear ultrasound probe is placed in the midaxillary line and the fifth intercostal region in the sagittal plane (Figure 3A). The latissimus dorsi, teres major, and serratus muscles are visualized on the ribs and pleura. Visualization of the thoracodorsal artery helps define the serratus muscle superficial plane, and local anesthetic is injected into this plane for superficial SAP block. In the deep SAP block, the injection is performed between the serratus anterior muscle and the costal periosteum (Figure 3B).

Considering the block application area, the SAP block is suitable for minimally invasive cardiac surgeries accompanied by thoracotomy. These surgeries have been reported to be associated with a decrease in both pain scores and opioid requirements when compared to the control group.^40,41^ A hundred adults who underwent heart surgery via thoracotomy by Magoon et al^24^ were randomly assigned to the intercostal nerve block, PSP block, or SAP block groups. They reported that while all 3 groups’ early pain scores were same, the SAP and PSP blocks’ late mean pain scores were considerably lower. Compared to the SAP and PSP block groups, the intercostal group required more rescue fentanyl.

### Erector Spinae Plane Block

This block, performed on the posterior chest wall, is called the ESP block since the local anesthetic injection is performed between the erector spinae muscle and the periosteum of the vertebral transverse process. Although the targeted dermatomal area varies according to the application area, it can include the ventral branches as well as the dorsal branches of the T2-T6 intercostal nerves.^42^ The ESP block should be applied in the sitting or prone position and before the incision. 

**Figure 4. A,B.**
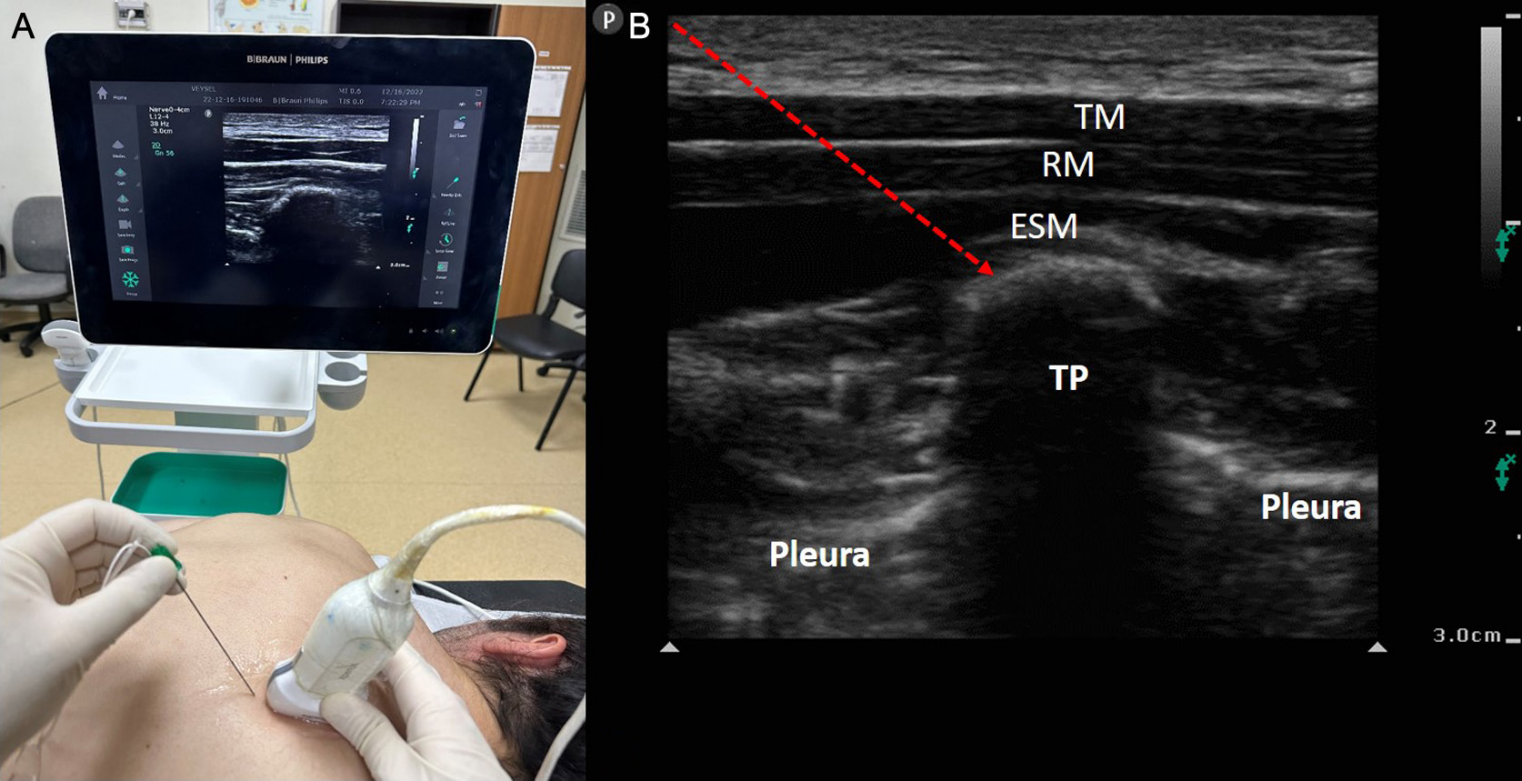
(A) Patient and ultrasound probe position for thoracic ESP block procedure. (B) Sonographic anatomy of the block. ESM, erector spinae muscle; red arrow, needle; RM, rhomboid muscle; TM, trapezius muscle; TP, transverse process.

In the ESP block procedure, the linear ultrasound probe is placed in a longitudinal orientation 3 cm lateral to the fourth thoracic vertebral spinous process (Figure 4A). A hyperechoic transverse process is visualized under the trapezius, rhomboid major, and erector spinae muscles. An ESP block is performed with the injection of local anesthetic between the erector spinae muscle and the transverse process (Figure 4B). It is confirmed by the spread of the local anesthetic throughout the plane.

Fifty patients undergoing heart surgery were randomly assigned to the thoracic epidural groups and bilateral continuous ESP by Nagaraja et al.^43^ The day before the procedure, both interventions were administered. The researchers showed that ICU length of stay, incentive spirometry, and mechanical ventilation were all similar. The mean scores were lower than 4/10 for both groups, despite the fact that the pain scores varied significantly.

About 106 patients undergoing elective heart surgery who required cardiopulmonary bypass (CPB) were randomly assigned to either the ESP or the acetaminophen and tramadol groups by Krishna et al.^26 ^Patients in the ESP group reported considerably less pain overall, and their analgesia lasted noticeably longer. The ESP group was extubated, able to tolerate nutrition, and discharged from the ICU earlier. As a result, the ESP block’s improved analgesia not only assisted with pain scores but also produced quantifiable results that enhanced the success of these surgeries.

Besides, Dost et al^44^ indicated that adding a superficial PIP block to the ESP block reduced the postoperative morphine requirement and pain scores in patients undergoing cardiac surgery under median sternotomy. In a recent meta-analysis, the authors found that when comparing 24-hour morphine milligram equivalents, fascial plane blocks were superior to placebo.^45^

## Overall Perspective

The field of cardiac surgery lags behind as we advance toward ERAS and opioid-free anesthesia. In the perioperative period, the conventional severe narcotic treatment is related to nausea, urine retention, delayed extubation, and numerous pulmonary difficulties. The optimum analgesia for surgery is provided by neuraxial procedures such thoracic epidural and even purposefully high spinal levels; however, anesthetists are typically cautious of potential side effects such severe hypotension and epidural hematoma. Hypotension from neuraxial techniques may exacerbate ischemia in patients with severe left main or triple vessel disease that depend on high blood pressure for coronary perfusion. Additionally, anesthesiologists may not always want to undertake neuraxial procedures on these patients because they frequently receive heparin infusions before to surgery and will be extensively heparinized during it.

Regional nerve blocks offer a successful method for enhancing pain management for heart surgery patients in an era where knowledge of the risks of abusing opioid-based anesthesia and analgesia is growing. The adaptability of regional analgesia, which until recently was primarily restricted to neuraxial alternatives in the cardiac surgery environment, has increased as a result of developments in recent years. Regional analgesia for cardiac surgery is still in its infancy, although showing promise. Most of the regional techniques were defined by observational studies with small sample sizes and studies compared with control groups without regional intervention. Variable block timing, indwelling catheters, and a wide range of injectables and ultrasound techniques are all used in the studies. Peripheral nerve blocks are generally considered safe. In a retrospective analysis of 70 patients undergoing mitral valve surgery did not report any problems with coagulation.^46^ Due to the relative frequency of problems, the majority of studies lack the necessary power to establish safety profiles. Due to the lack of historical context of cardiac surgery procedures in terms of regional anesthetic techniques, most institutions do not have the equipment necessary for regional anesthesia interventions, the clinical experience and monitoring unit necessary to consistently run a comprehensive program. As a result of these factors, there are still obstacles to the widespread use of localized analgesia in cardiac surgery and is a sign that the literature on this subject needs to be developed further.

## Abdominal Surgery

Before regional anesthesia interventions under ultrasound, it is necessary to master the anatomy of the anterior-lateral wall of the abdomen and the upper abdomen. A significant part of the peripheral nerves, which originate from the spinal cord of the medulla and distribute to the body through the vertebral foramen, continue their course by passing through the interfascial areas.

When evaluated in the anterior to lateral direction on the anterior abdominal wall, rectus abdominis muscle and external oblique in 3 layers, internal oblique, and transversus abdominis muscles. Apart from these, regional anesthesia practitioners need to recognize the quadratus lumborum muscle, pulp major, and latissimus dorsi muscles, which are adjacent to the vertebral area and displayed in regional anesthesia procedures applied for the lower and upper anterior abdominal wall.^47,48^

Peripheral nerves originating between T6 and T12 are divided into 2: anterior and posterior ramus at first, then they are divided into further 2, anterior ramus and anterior and lateral cutaneous branches. The lateral cutaneous branch continues between the innermost and internal intercostal muscles as T6-T8 intercostal nerves, the anterior cutaneous branch (T6-T11) passes the costal margin after its passage in the intercostal space, travels between the quadratus lumborum and psoas major muscles, and as a result, they continue between the anterior abdominal wall muscles. These nerves are called thoracoabdominal nerves. During this passage, some peripheral nerves emerging from T6-12 receive the somatic sensation of the lateral abdominal wall and parietal peritoneum at the level of the anterior and midaxillary line of the abdomen, and travel between the internal oblique and transversus abdominis muscles, several peripheral nerves originating between T7-12 travel between the rectus abdominis muscle and rectus sheath for the somatic sensation of the anterior abdominal wall. It runs between the internal oblique and transversus abdominis muscle in the ilioinguinal and iliohypogastric nerve, which originates from T12 and L1 and carries the sensation of muscle, skin, and parietal peritoneum throughout the peri-inguinal and inguinal region.^49,50^

### Transversus Abdominis Plane Block

It was first defined by Rami et al in 2001 using the petit triangle as a landmark. With the introduction of ultrasound in anesthesia practice, there are different approaches to the anterior abdominal wall, such as intercostal/subcostal, oblique subcostal, bilateral dual-transversus abdominis plane (TAP), lateral/classical, anterior, and posterior. In the TAP block application, a blocking medication is applied between the internal oblique muscle and the transversus abdominis muscle. Since it is affected by individual anatomical variations, different dermatomal involvements have been observed in the literature with cadaveric studies, radiological imaging, and physical examination. It is used in the clinic for abdominal surgeries (abdominoplasty, hysterectomy, colorectal surgery, laparoscopic abdominal surgeries, cesarean, midline laparotomy, bariatric surgery), urological procedures (renal transplantation, nephrectomies, prostatectomies, varicocelectomy), and genitourinary surgery.

**Figure 5. A,B.**
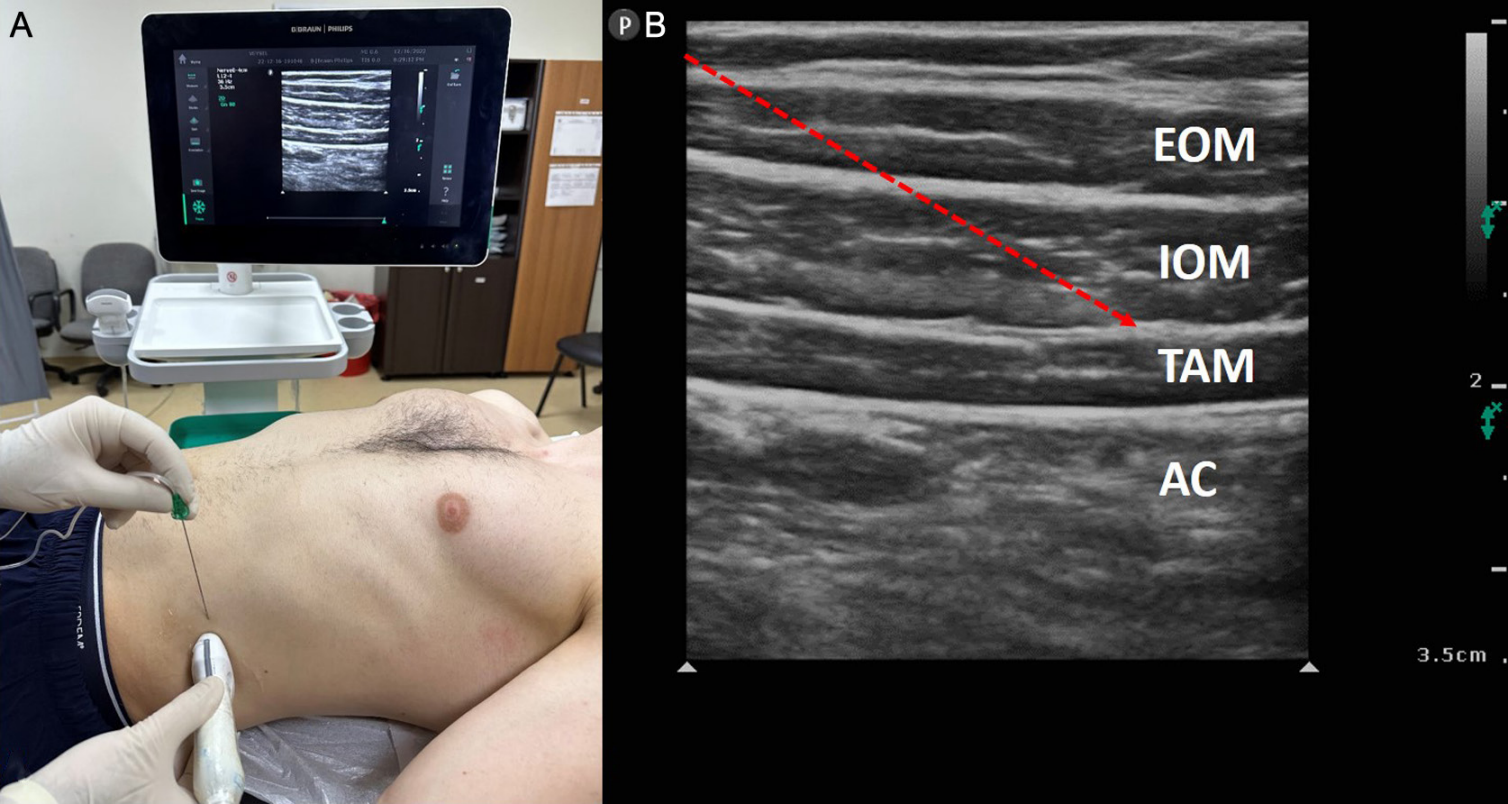
(A) Patient and ultrasound probe position for transversus abdominis plane block procedure. (B) Sonographic anatomy of the block. AC: abdominal cavity; EOM, external oblique muscle; IOM, internal oblique muscle; red arrow, needle; TAM, transversus abdominis muscle.

Many different approaches for TAP block applications have been described by the practitioners. It has been demonstrated in the clinical trials that mainly the dermatomes between T6 and T9 are affected by the* subcostal approach *applied by placing the USG probe lateral to the xiphoid process parallel to the costal margin, dermatomes between T6 and L1 by the oblique subcostal approach applied by placing the probe between the midclavicular and anterior axillary lines parallel to the costal margin, dermatomes between T10-T12 by the *lateral approach* applied by placing the probe on the midaxillary line in a parallel manner to the superior of the iliac crest, dermatomes between T7 and T12 by the bilateral dual-TAP approach as a combination of bilateral subcostal and lateral TAP blocks, and dermatomes between T9 and T12 by the posterior approach applied by placing the probe on the posterior axillary line parallel to the superior of the iliac crest (Figure 5). Hence, it was revealed that the efficiency is increased by applying TAP blocks from different anatomical sites for different indications.^51^

When clinical applications are evaluated, besides single-injection TAP techniques, there are TAP block applications for analgesic purposes in the form of continuous intermittent injections with a pain pump or intermittent injections without the need for a pump. There are many studies in the literature evaluating the analgesic effectiveness, analgesic consumption, patient satisfaction, side effects, and incidence rates of ESP, quadratus lumborum block (QLB), Ilioinguinal iliohypogastric (IL-IH), wound infiltration, and TAP blocks. In addition, there are studies comparing epidural, caudal, paravertebral blocks, and Patient controlled analgesia (PCA) devices, and patients were given opioids. In all studies, the significant superiority of all these blocks to each other could not be demonstrated.^52-54^

### Rectus Sheath Block

**Figure 6. A,B.**
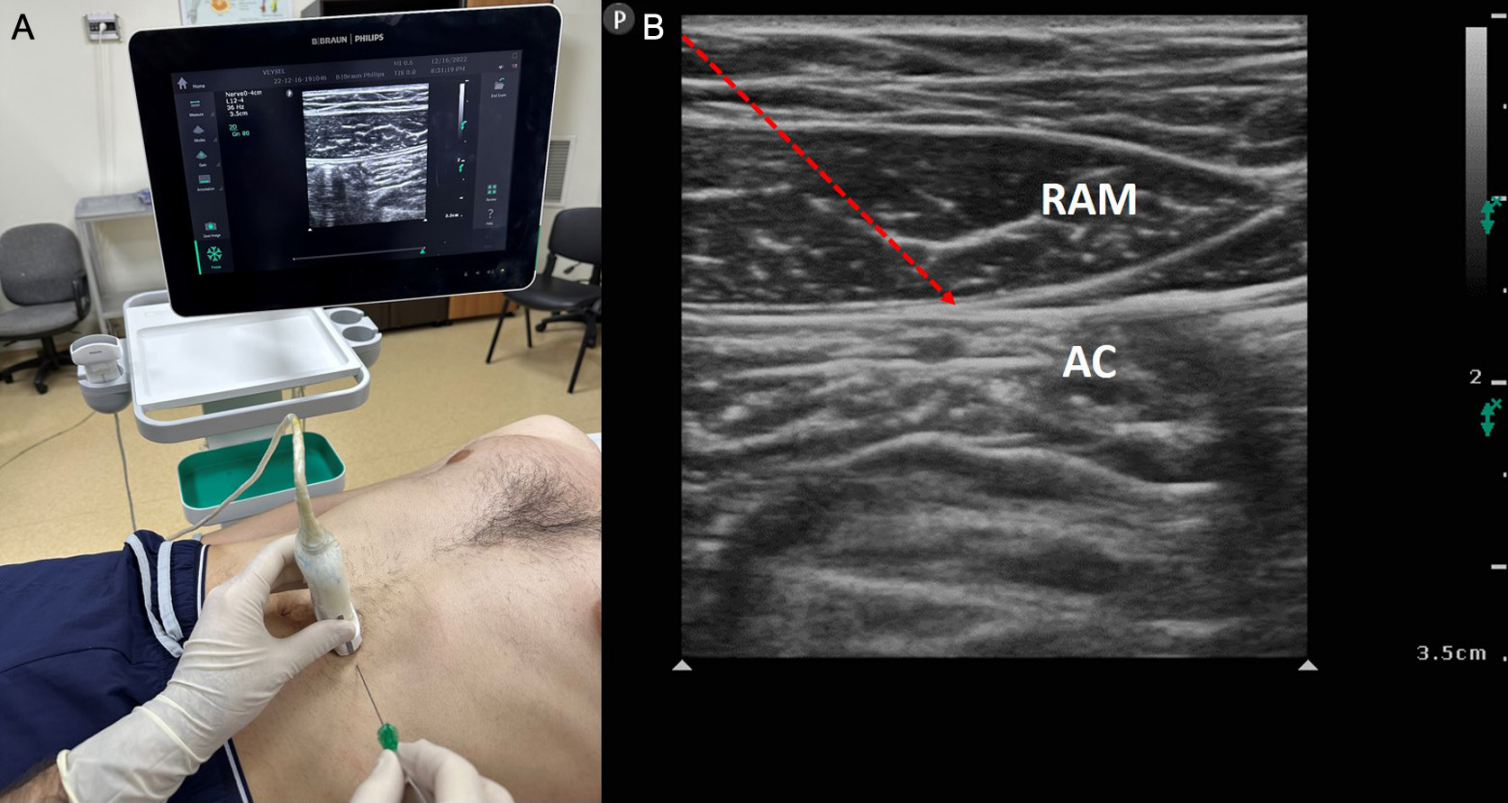
(A) Patient and ultrasound probe position for rectus sheath block procedure. (B) Sonographic anatomy of the block. AC, abdominal cavity; RAM, rectus abdominis muscle; red arrow, needle.

The rectus sheath block was historically described in the early 20th century and is still popularly used in mid-abdominal wall interventions today. In the rectus sheath block application, block medication is applied between the rectus muscle and its posterior sheath (Figure 6). There are 4 different quadrant applications, bilaterally in the upper and lower quadrants of the umbilicus, or different rectus sheath block applications in which the catheter application is defined bilaterally. The target for rectus sheath block (RSB) is the terminal peripheral nerve endings of the T7-T12 spinal nerves that come to the mid-abdominal wall. It has been frequently used in the last decade to reduce postoperative pain secondary to trocar incisions, especially after laparoscopic surgery. When the literature is reviewed, better pain control, less opioid consumption, and fewer side effects secondary to opioids were observed in the postoperative period compared to the control groups.^55^

There are many studies in which the rectus sheath block has been used with a single block or a combined block with different blocks. As a result of meta-analyses evaluating especially laparoscopic and laparoscopic abdominal surgeries, it was observed that pain scores decreased and patient satisfaction increased during the first 12 hours after surgery. Besides, a meta-analysis evaluating the effectiveness of RSBs in the pediatric age group reported that they provided effective analgesia.^56,57^In gynecological cancer surgery, there are studies where no significant difference was observed between groups in pain scores when compared to epidural anesthesia between patient groups who underwent continuous epidural and continuous rectus sheath block catheter and between patient groups who underwent colorectal surgery.^58,59^ Furthermore, there are different case series in the literature, including gynecological, colorectal, and inguinal hernia repair surgeries, along with complementary analgesic techniques for the anterior and lateral walls of the abdomen, such as TAP block.^60^

### Quadratus Lumborum Plane Block

TAP has been described more recently than the block. Its effectiveness was defined by the block agent applied around the quadratus lumborum muscle, located between the thoracolumbar fascia and the lumbar vertebral area, which is anatomically surrounded by the external oblique, internal oblique, and transversus abdominis muscles. In the literature, there are many studies stating that TAP block shows more effective analgesia because it covers a narrower area compared to the application area.

**Figure 7. A,B.**
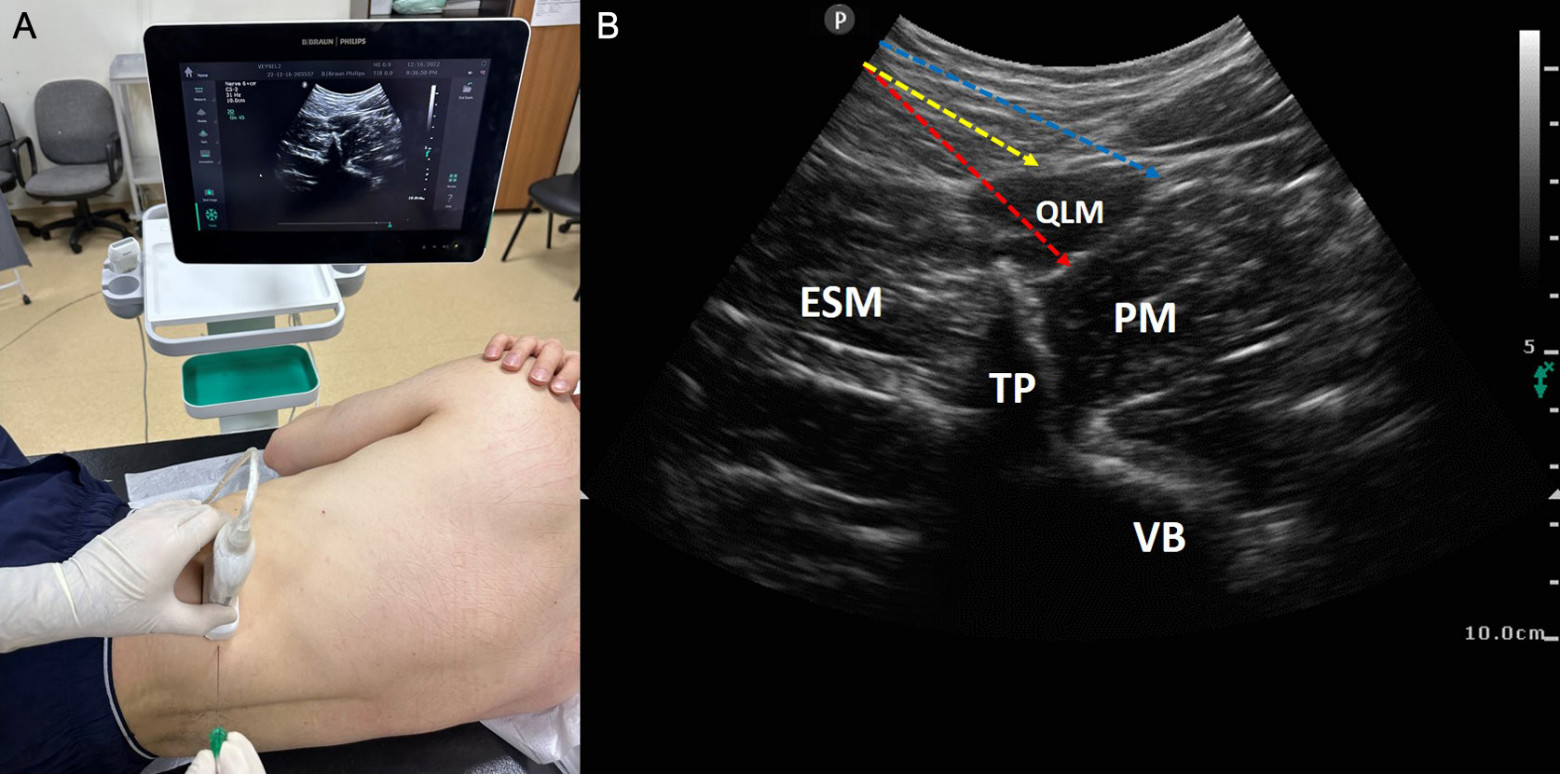
(A) Patient and ultrasound probe position for quadratus lumborum plane block procedure. (B) Sonographic anatomy of the block. Blue arrow, needle direction for lateral quadratus lumborum block; ESM, erector spinae muscle; PM, psoas muscle; QLM, quadratus lumborum muscle; red arrow, needle direction for anterior quadratus lumborum block; TP, transverse proses; VB, vertebral body; yellow arrow, needle direction for posterior quadratus lumborum block.

Different approaches for QLB have been described by practitioners (Figure 7). QLB-1 is defined as the application into the thoracolumbar fascia between the lateral side of the quadratus lumborum muscle (QLM) and the endpoint of the transversus abdominis muscle, QLB-2 (posterior approach) as the application to the posterior surface of the QLM between the investing layer of the thoracolumbar fascia, QLB-3 (transmuscular approach, anterior approach) as the application to the anterior surface of the QLM muscle and between the anterior surface of the psoas major muscle, and QLB-4 (intermuscular approach) as the application to the inside of the QLM. Since the QLM extends caudally with the psoas major and iliacus muscle, it can show truncal block characteristics and may also form a lumbar plexus block by spreading to the lumbar plexus. This feature has also been demonstrated by studies describing its analgesic efficacy in hip surgeries.^61^

When clinical applications are evaluated, there are single-injection QLB techniques, as well as QLB block applications for analgesic purposes in the form of catheters and pain pumps or intermittent injections. Case series and similar randomized controlled studies in which QLB catheter applications, including donor nephrectomy, nephrolithotomy, spinal fusion, laparotomic abdominal surgeries, laparoscopic colorectal surgeries, cesarean and hip surgeries, have been performed, as well as providing somatic analgesia with single-injection techniques, especially by blocking the thoracolumbar nerves are reported.^62-64^. There are many studies in the literature evaluating the analgesic effectiveness, analgesic consumption, patient satisfaction, side effects, and incidence rates between central blocks and ESP, TAP, wound infiltration, and QLB blocks. Erector spinae plane block applied from the thoracic and L1 level and posterior approach QLB applied liver resection, donor nephrectomy surgeries, and lower abdominal surgery were not demonstrated to be superior to each other. Although studies have revealed an increase in analgesia duration and analgesia quality with QLB block compared to TAP block in lower segment cesarean surgeries and inguinal hernia surgeries, there are studies with similar efficacy.^63-66^ Extracorporeal shock wave lithotripsy (ESWL), one of the daily applications, showed that the fentanyl consumption of the patients was significantly lower than the control group, and the fragmentation rates were significantly higher than the control group.^67,68^ In a study evaluating the efficacy of QLB-2 and QLB-3 blocks in open inguinal surgeries, it was observed that both blocks reduced opioid consumption and extended first analgesia times in the patient group compared to the control group. It was determined that opioid consumption in the QLB-3 group was significantly lower in the patient group in which the block was applied compared to the QLB-2 group, except for the first 4 hours in the first 24 hours.^69^

### Transversalis Fascia Plane Block

**Figure 8. A,B.**
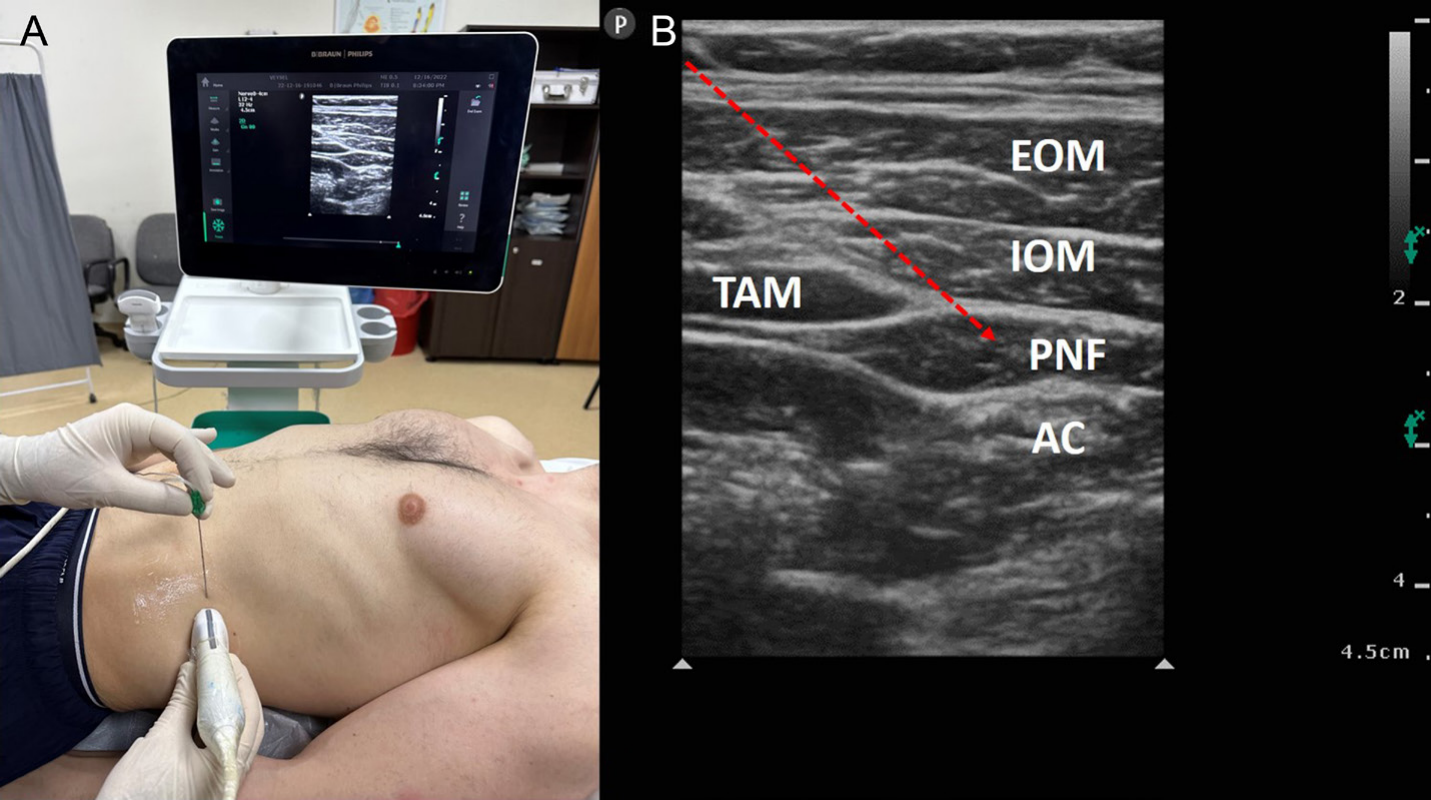
(A) Patient and ultrasound probe position for transversalis fascia plane block procedure. (B) Sonographic anatomy of the block. AC: abdominal cavity; EOM, external oblique muscle; IOM, internal oblique muscle; PNF, perirenal fat tissue; red arrow: needle; TAM, transversus abdominis muscle.

It was first described in 2009 by blocking the cutaneous branches and terminal ends of the T12 and L1 nerves. In addition to the inguinal and peri-inguinal area that is the target for the block, studies are showing its effectiveness in iliac crest grafts and hip surgery. It is necessary to define the abdominal cavity and peritoneum, the transversus abdominis muscle, and the perirenal adipose tissue for application. It shows effectiveness with the block medication applied into the perirenal adipose tissue during the passage to T12 and L1 origin peripheral nerves through the perirenal adipose tissue (Figure 8). Significant efficacy has been demonstrated in the literature for inguinal hernia surgery, appendectomy, varicocelectomy, cesarean section surgery, hip surgery, and iliac crest harvesting.^70,71^

Since the block targets T12-L1 nerves, their clinical use is mostly compared to iliohypogastric-ilioinguinal nerve blocks and wound infiltrations, as well as transversalis fascia plane (TFP) block applications in iliac crest harvesting surgeries due to close anatomical areas. Although there are studies showing that inguinal hernia repair surgery creates a more effective analgesic effect than transmuscular-posterior QLB application, there are also studies indicating no difference between them in terms of anesthetic effectiveness and duration.^66,72^ A group of patients who underwent developmental dysplasia of the hip undergoing open reduction surgeries demonstrated that TFP block delayed the first analgesia requirement time and significantly reduced total analgesic consumption.^73^ In cesarean surgery, it was determined that compared to the patient group in which spinal anesthesia and TFP block were applied and spinal anesthesia combined with placebo (saline) TFP block, they consumed less morphine, nausea-vomiting was less common, and patient satisfaction was higher.^74^ In the case series of 5 patients who underwent pediatric ureteroneocystostomy surgery, it was reported in the study that FLACC scores did not exceed 3 in the first 24 hours.^75^ Again, in the case series of pediatric abdominal surgery in 2 patients, it was observed that FLACC scores were below 3, and the first analgesia requirement was at the 16th hour.^76^

### Thoracoabdominal Nerve Block Perichondrial Approach

**Figure 9. A,B.**
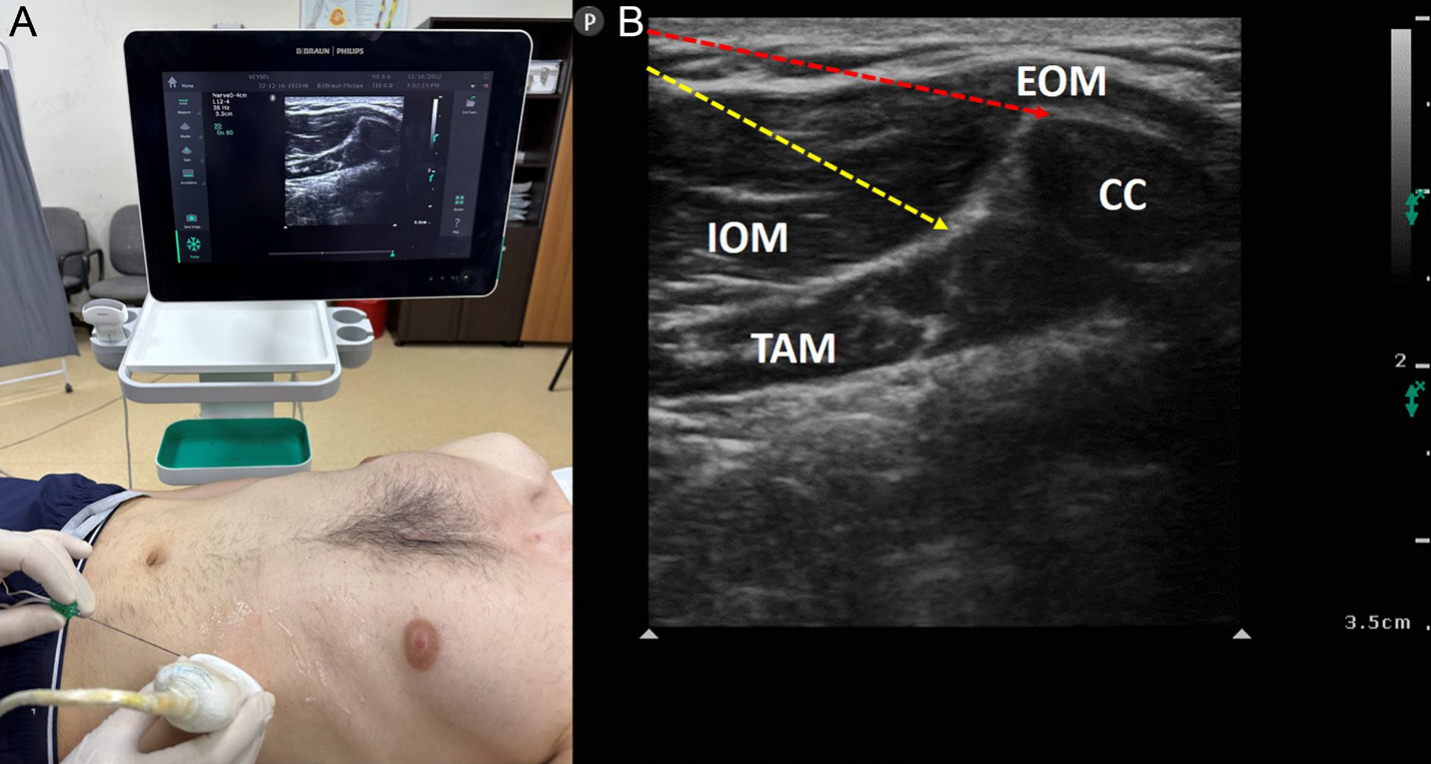
(A) Patient and ultrasound probe position for thoracoabdominal nerve block perichondrial approach procedure. (B) Sonographic anatomy of the block. CC, costal cartilage; EOM, external oblique muscle; IOM, internal oblique muscle; TAM, transversus abdominis muscle; arrows: needle direction. Red and yellow arrow: injection points for TAPA block, yellow arrow: injection point for mTAPA block.

Tulgar described this approach in 2019. It is mainly used in surgeries at the abdominal and thoracoabdominal junction by affecting the lateral and anterior branches of the thoracoabdominal nerves. Since it is challenging to perform using conventional techniques, it is preferably applied under ultrasound guidance.^77^ Studies have demonstrated its effectiveness in transcatheter aortic valve implantation and abdominal surgery, including laparoscopic cholecystectomy, laparoscopic distal gastrectomy, and laparoscopic ventral hernia repair surgeries. It is applied with a linear ultrasound probe placed sagittally on the costochondral junction. As a result of the block, it was observed that the dermatomal area involved was T5-T12.^78^ For the TAPA block, block medication is applied between the internal oblique and transversus abdominis muscle, like the TAP block, under the rib, seen at the costochondral junction and between the external oblique muscle and intercostal muscle on the rib (Figure 9). In the modified TAPA block, in addition to the TAPA block application, the block agent is applied between the internal oblique and transversus abdominis muscles, as in the TAP block. Some studies suggest that M-TAPA is administered as a single injection and may be more suitable for abdominal-weighted interventions. A cadaver study revealed that only T8-T11 fibers were affected during the m-TAPA block, despite 2 different volumes of 25 mL and 30 mL.^79^ However, there are clinical studies reporting dermatomal involvement between T5-T10 and T3-T12 with 20 mL of 0.25% bupivacaine-containing block medication.^80,81^

### External Oblique Intercostal Block

In contrast to the previously defined blocks for abdominal interventions, different block definitions for upper and lower abdominal analgesia have been defined by many clinicians in the last decade. It is applied by the practitioners who prioritize analgesia in the external oblique intercostal block and upper abdominal interventions such as subcostal TAP, TAPA, and m-TAPA. Some clinical studies have demonstrated with case series that blocks of T5-T11 and T12 levels can be achieved with adequate medication.^82^ In addition, the external oblique intercostal block has been shown in cadaveric studies that the block medication can go up to T6 and above with its course behind the latissimus dorsi and serratus anterior muscles.^83^

**Figure 10. A,B.**
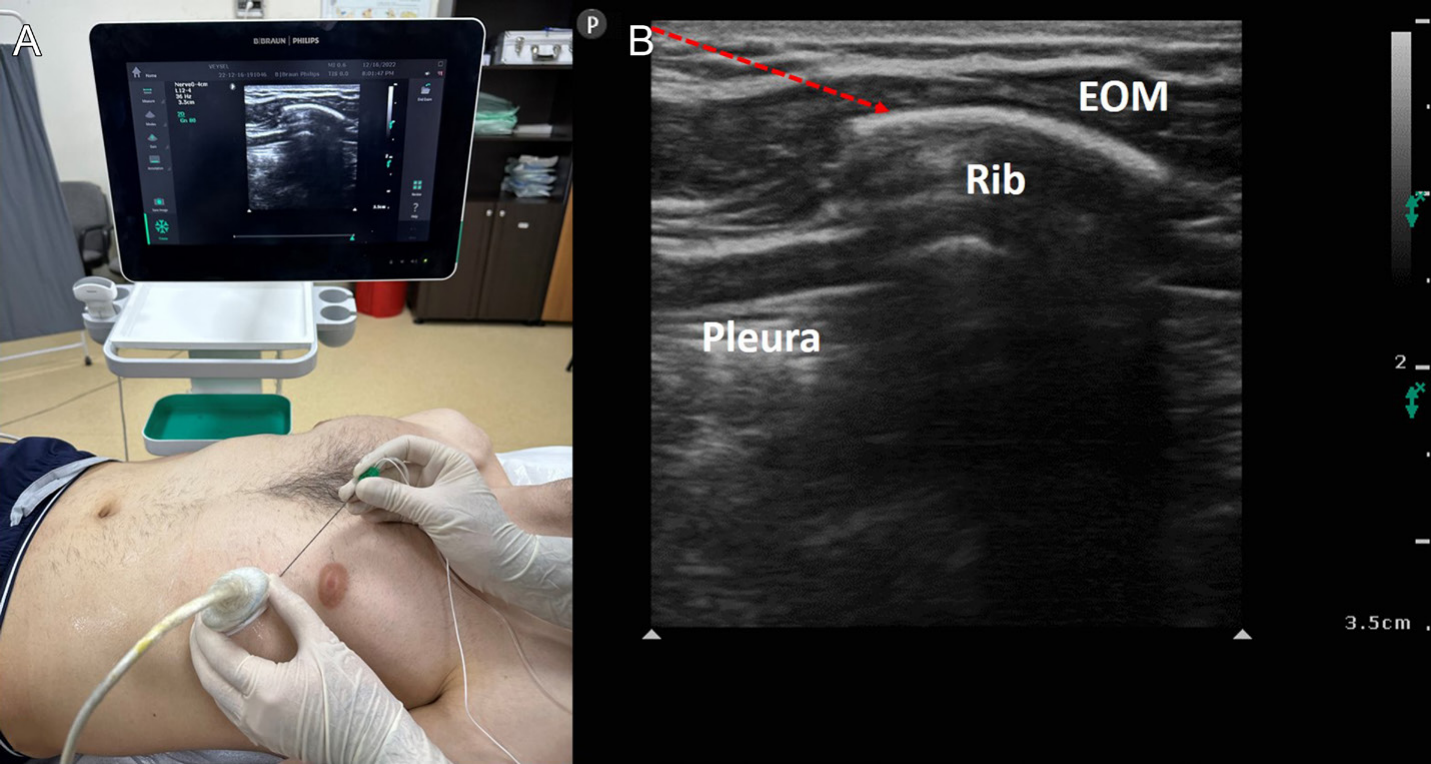
(A) Patient and ultrasound probe position for external oblique intercostal block procedure. (B) Sonographic anatomy of the block. EOM, external oblique muscle; red arrow, needle direction.

To apply the external oblique intercostal block, it is crucial to define the sixth and seventh ribs on the anterior axillary line under ultrasound. The block needle is applied by directing it under the external oblique muscle in the cephalic to the caudal position (Figure 10). Although there are no randomized controlled studies in the literature, there are few case reports.^84^

### Spinal Surgery

Pain after spinal surgery is quite common compared to other types of operations. Spinal surgery usually involves a deep midline incision and retraction of the paraspinal muscles. During this surgery, bone, ligaments, durometer, facet joint, muscle, fascia, and cutaneous tissue are the main sources of pain. This situation causes severe post-surgical pain and patient discomfort in the postoperative period. Before the definition of interfascial plane blocks, there were no options for spinal surgery other than neuraxial/paravertebral techniques and wound infiltration. Fascial plane blocks used for spinal surgery can provide adequate analgesic activity by targeting the dorsal ramus of spinal nerves.

### Erector Spinae Plane Block

**Figure 11. A,B.**
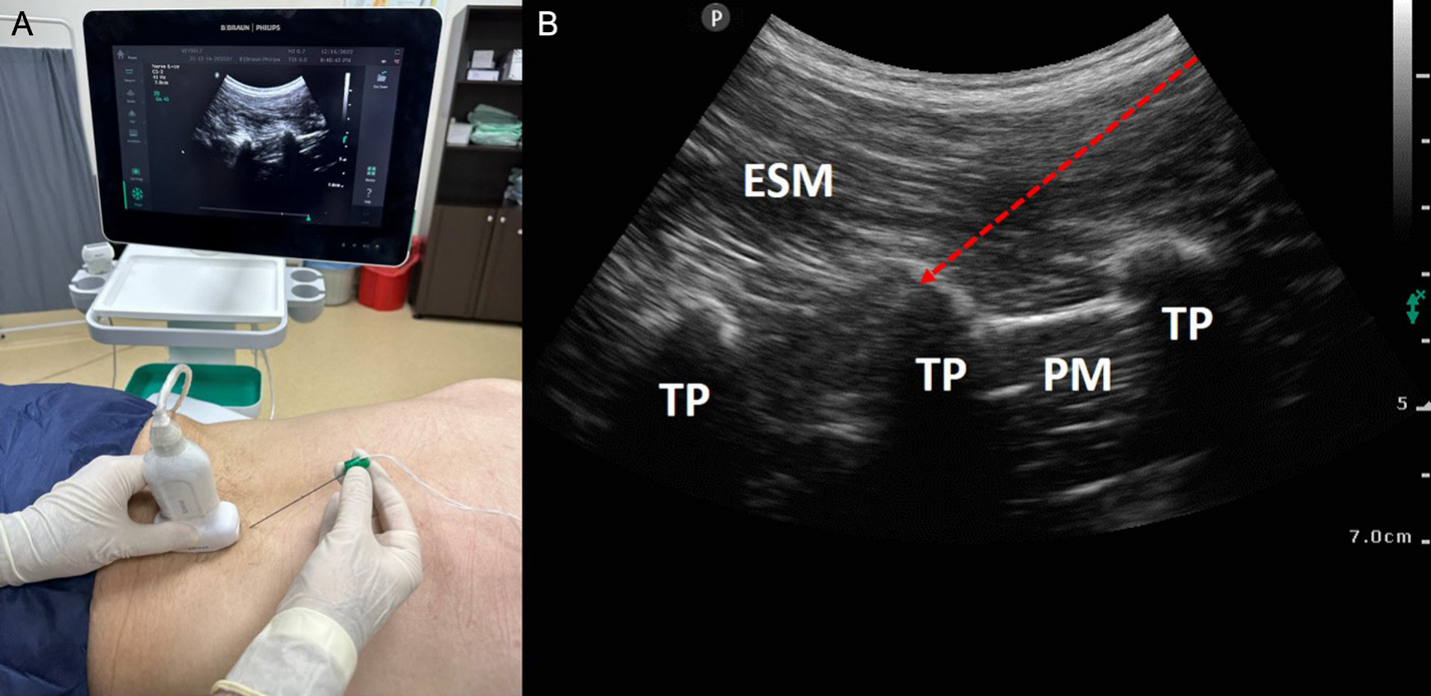
(A) Patient and ultrasound probe position for lumbar ESP block procedure. (B) Sonographic anatomy of the block. ESM, erector spinae muscle; PM: psoas muscle; red arrow: needle; TP, transverse proses.

After its first description in 2016, ESP block has been a game changer in regional anesthesia practice for many anesthesiologists worldwide due to its safety and technical simplicity. Since the erector spinae muscle is located in the cervical to sacral region, ESP block techniques have been developed and used for the entire vertebral column. The target tips for ESP blcok are between the vertebral transverse process and the erector spinae muscle (Figure 11). Efficiency in ESP block is considered to be due to its transition to the paravertebral area through the cruveilhier ligament on the costotransverse ligament or through the intertransverse connective tissue complex.^85^ The formation of both visceral and somatic analgesia after the application indicates that the block medication is transferred to the paravertebral, epidural, or spinal area. The block solution not only remains in the application area or passes through the anterior part of the vertebral column but also spreads in the cranial-caudal direction and the medial-lateral area. This spread can also explain the reason why the upper and lower dermatome areas are involved.

Erector spinae plane block can be applied with positions such as prone, sitting position, and lateral decubitus position. In the application of the block, the practitioners must have a good grasp of the anatomy of the different back regions, although it is necessary to define the 2 anatomical structures very well. This anatomy knowledge is crucial, especially to avoid complications such as paravertebral and epidural applications and pneumothorax. ESPB block can be applied in 2 different ultrasound positions, parasagittal and transverse, when the literature is evaluated. At the same time, the linear or convex probe is used with the preference of the practitioner. Parasagittal placement of the ultrasound probe is more beneficial to see the spread of the block solution. In addition to these data, in-plane or out-of-plane interventions are available in the literature. For thoracic ESP block application, the trapezius muscle, rhomboid major muscle, and erector spinae muscle at the T5 level in the superior to inferior direction are the trapezius muscle and erector spinae muscles at the T7 level. There is an erector spinae muscle at the L2 level for lumbar region ESP block application. In ESP block application, although the blocking medication applied under the erector spinae muscle is sufficient, targeting the transverse process to avoid an unintended paravertebral and pleural puncture, good follow-up of the block needle in the ultrasound field, and the successful block is appropriate to prevent unwanted complications.

Because ESP block contains visceral and somatic analgesic components, it has been used as both anesthetic and analgesic in pain palliation and many surgeries. Although there are randomized controlled studies in many surgeries such as neuropathic pain, shoulder pains, extremity complex regional pain syndrome and herpes zoster, laparoscopic cholecystectomy, breast surgery, cardiac surgery, thoracotomy, and lumber spine surgery, there are case reports of more than one hundred successful ESP blocks in the literature.

A study evaluating the efficacy of bilateral ESP block in lumbar spine surgery revealed that ESP block decreased 24-hour morphine consumption compared to the control group and reduced pain scores with rest and movement.^86^ Similarly, another study demonstrated no difference between the control group and ESPB block for pain scores alone, which reduced short-term opioid consumption compared to wound infiltration.^87^ In a study performed in lumbar spinal decompression surgery, it was reported that bilateral ESP block reduced pain scores, decreased tramadol consumption, and prolonged the time to first analgesic consumption compared to the control group.^88^ A meta-analysis study evaluating lumbar surgeries determined that ESP block significantly reduces pain scores and analgesic consumption.^89^ In a study evaluating single-shot ESP block surgeries, including lumbar surgeries, compared to control groups, a meta-analysis similarly showed that opioid consumption was low, first rescue analgesic times were later after surgery, and rescue analgesic consumption was low.^90^

### Thoracolumbar Interfascial Plane Block

**Figure 12. A-D.**
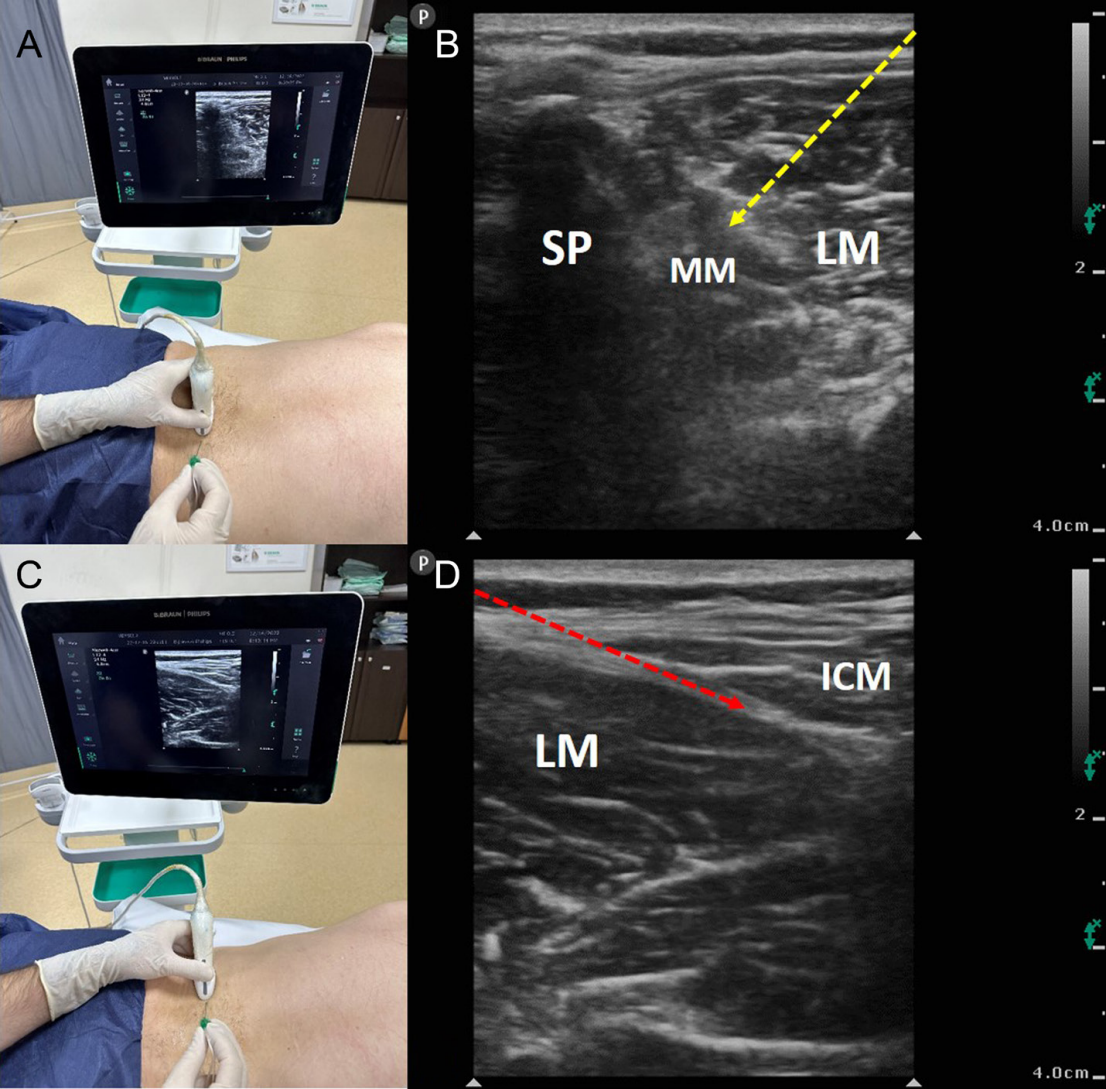
(A, C) Patient and ultrasound probe position for lumbar thoracolumbar interfascial plane block procedure. (B, D) Sonographic anatomy of the block. ICM, iliocostalis muscle; LM, longissimus muscle; MM, multifidus muscle; red arrow: needle direction for modified TLIP block; SP, spinous process; yellow arrow: needle direction for TLIP block.

Thoracolumbar interfascial plane block (TLIPB) was described by Hand et al in 2015.^91^ The erector spinae muscle consists of 3 muscles. These muscles are the medial to lateral multifidus, longissimus, and iliocostalis muscles. After the nerves emerging from the intervertebral foramen divide into dorsal and ventral branches, the dorsal ramus gives way to the periphery by giving muscular and cutaneous branches in the posterior half of the body. Since the peripheral nerves originating from the dorsal ramus travel through the erector spinae muscles, the blockage of the dorsal ramus is targeted with TLIPB with the blocking agent applied between the multifidus and longissimus muscles (Figure 12A and B). Thoracolumbar interfascial plane block was modified by Ahiskalioglu et al^92^ and a different approach was developed as mTLIPB with the application between the longissimus and iliocostalis muscles (Figure 12C and D). While both blocks are applied, the ultrasound probe is placed transversely and shifted laterally on the L3 processus spinosus vertebra. In addition to the difference between the application areas between the 2 blocks, there are also some application differences.^93^ While TLIPB is applied, the block needle is oriented lateral to medial, while mTLIPB is oriented medially to lateral. This maneuver facilitates the placement of the block needle in the interfascial area. Difficulty in evaluating the multifidus muscle under ultrasound can be a handicap for TLIPB.

When TLIPB and mTLIP are evaluated in the literature, they are determined to be frequently used in lumbar spinal surgeries. A study evaluating the efficacy of TLIPB in posterior spine fusion surgery revealed that the time of first rescue analgesia was statistically delayed in patients treated with TLIPB compared to the control group and the infiltration group.^94^ A meta-analysis study evaluating the postoperative analgesic efficacy of TLIPB in lumbar spine surgery indicated that TLIPB significantly reduced pain at rest and movement in the postoperative period and reduced PCA consumption.^95^ In posterior lumbar decompression and stabilization surgery patients comparing TLIPB and mTLIPB with postoperative pain and IL-6 level, it was observed that IL-6 level and numeric rating scale averages were lower in the mTLIPB group. However, there was no difference in Qnor values and total morphine consumption.^96^ A study comparing the applicability and analgesic efficacy of TLIPB and mTLIPB in lumbar disc surgery has shown that the applicability of mTLIPB is faster, analgesic efficacy, opioid consumption, and rescue analgesic consumption are similar.^97^ In a study evaluating the analgesic efficacy of epidural anesthesia applied during closure in lumbar discectomy surgery and mTLIPB applied before surgery, while there was no difference in opioid consumption in the first 4 hours after surgery, there was a significant decrease in favor of mTLIPB between the 8th and 24th hours in terms of opioid consumption.^98^
